# Complete plastome sequence of *Acorus tatarinowii* (Acoraceae), a traditional Chinese medicinal plant from Xishuangbanna, Yunnan, China

**DOI:** 10.1080/23802359.2019.1694852

**Published:** 2019-12-13

**Authors:** Gong Yan-Xiong, Tian Yao-Hua, Nan Jiang, Wen-Bin Yu

**Affiliations:** a Yunnan Institute of Tropical Crops, Jinghong, China;; b Center for Integrative Conservation and Southeast Asia Biodiversity Research Institute, Xishuangbanna Tropical Botanical Garden, Chinese Academy of Sciences, Mengla, China;; c Center of Conservation Biology, Core Botanical Gardens, Chinese Academy of Sciences, Mengla, China

**Keywords:** *Acorus tatarinowii*, chloroplast genome, Acoraceae

## Abstract

The complete plastome of *Acorus tatarinowii* is 153,296 bp in length, with two long inverted repeats (25,752 bp for each) separating by a large single-copy (83,533 bp) and a small single-copy (18,240 bp). The plastome contained 112 unique genes, including 78 protein-coding genes, 30 transfer RNAs, and 4 ribosomal RNAs. Phylogenetic analyses showed that *A. tatarinowii* was closely related to *A. gramineus.*

*Acorus tatarinowii* Schott (Acoraceae) is a perennial herb and widely distributed in southern China (Li 1979). It is an important traditional Chinese medicinal plant for treating heart, stomach and brain diseases (Lam et al. 2016; Lu 2017; Yang et al. 2017; Lam et al. 2019; Li et al. 2019). Moreover, plants of *A. tatarinowii* is also widely used by Dai medicine for treatments of watery diarrhea, asthma, headache and bloating (State Administration of Traditional Chinese Medicine 2005). To date, three *Acorus* species have sequenced the plastome, but *A. tatarinowii* was not investigated yet. Here, we reported a complete plastome sequence of *A. tatarinowii* in the first time, which will be used to develop DNA markers for molecular authentication and conservation genetics of *Acorus*.

Fresh leaves of *A. tatarinowii* was collected at Xishuangbanna Tropical Botanical Garden, Chinese Academy of Sciences (21°55′02.6″N 101°16′09.6″E), Mengla, south Yunnan, China, and frozen with liquid nitrogen. Genomic DNA was extracted using a modified CTAB method (Doyle and Doyle 1987). A voucher specimen (collection no. *YWB2019-021*) was deposited at Herbarium of Xishuangbanna Tropical Botanical Garden, Chinese Academy of Sciences (HITBC). The 150 bp pair-end reads were generated by Illumina NovaSeq 6000 using 350 bp insert-size library (Annoroad, Beijing). Around 12.98 Gb clean data with 35,189,804 reads were *de novo* assembled using GetOrganelle toolkit (Jin et al. 2018). The plastome was annotated using CPGAVAS2 (Shi et al. 2019), then manually adjusted in Geneious (Kearse et al. 2012). DNA polymorphism analysis was performed using DnaSP (Rozas et al. 2017).

The whole plastome of *A. tatarinowii* was 153,296 bp (MN536753) in size by having a large single-copy (LSC, 83,532 bp), a small single-copy (SSC, 18,240 bp), and two inverted repeats (IRs, 25,752 bp for each). The plastome contained 132 genes in total, including unique genes in 78 protein-coding, 30 tRNA, and 4 rRNA. There are five full and three partial protein-coding (*rps12*, *rps19*, and *ycf1*), eight tRNA and four rRNA genes in IRs. The overall GC content was 38.7%, and that of LSC, SSC, and IR regions were 37.3%, 33.3%, and 42.9%, respectively.

The whole sequences with one IR region of 42 taxa of angiosperms were aligned using MAFFT (Katoh and Standley 2013), then gaps were trimmed by trimAl (Capella-Gutiérrez et al. 2009) using the command ‘-gt 0.6 -cons 60’. For Maximum Likelihood analyses, we used RAxML (Stamatakis et al. 2008) using GTRGAMMAI model with 1000 bootstraps to reconstruct phylogeny of *A. tatarinowii*. Phylogenetic analysis showed that Acorales was the most basal order of monocots, and *A. tatarinowii* was sister to *A. gramineus* (Figure 1). DNA polymorphism analysis of four *Acorus* plastomes with one IR showed that there are 1302 variable and 601 parsimony-informative sites, respectively, and high variable and parsimony-informative regions occur at *trnT*^(UGA)^-*psbD*, *trnM*^(CAU)^-*atpE*, *ycf1*, and *trnL*^(UAG)^-*ndhF*, which are ideal regions for DNA barcodes of molecular authentication of *Acorus* spp. Moreover, the differences between *A. tatarinowii* and *A. gramineus* were 1971 bp, and between *A. tatarinowii* and *A. calamus*/*A. americanus* were 2087/2065 bp, which support *A. tatarinowii* to separate from *A. gramineus* as an independent species. Therefore, this new plastome sequence will be valuable for investigations on systematics and conservation genetics of *Acorus*.

**Figure 1.**
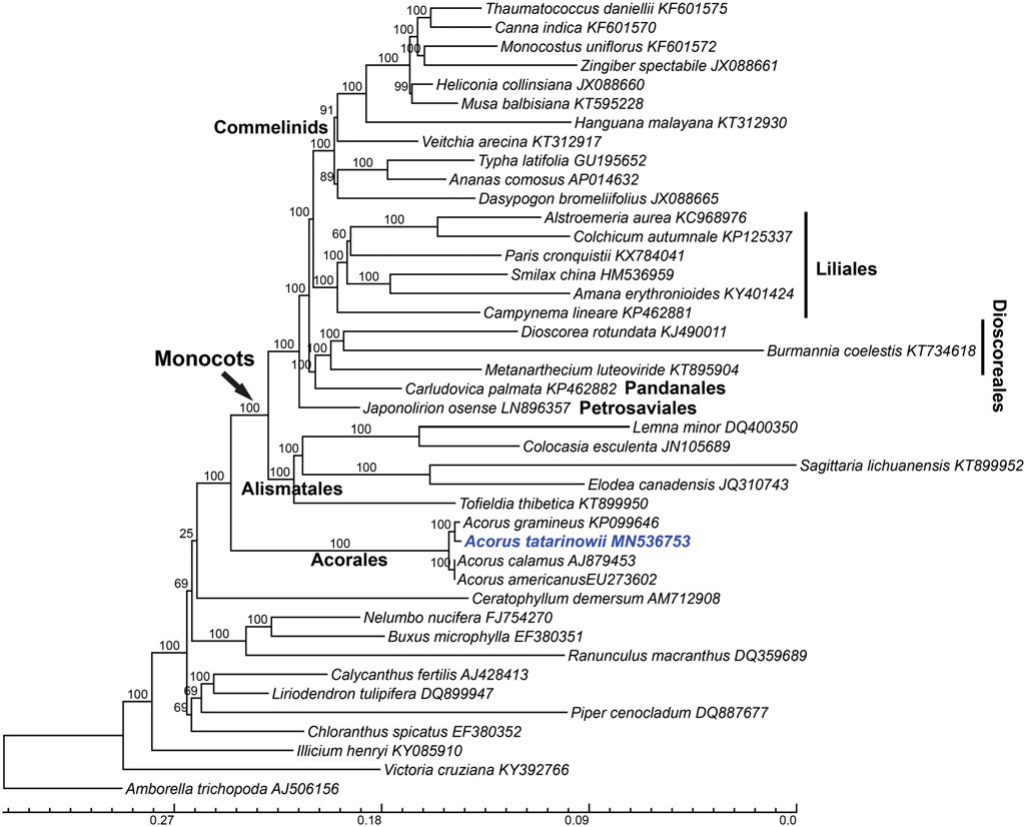
Plastome phylogeny of angiosperms based on Maximum-likelihood (ML) estimation. *Acorus tatarinowii* was highlighted by bold and blue style. ML bootstrap values of nodes indicated above the branch. The bottom scale bar represents the number of substitutions per site.
